# Expanding drug resistance through integron acquisition by IncFI plasmids of Salmonella enterica Typhimurium.

**DOI:** 10.3201/eid0703.010314

**Published:** 2001

**Authors:** A Carattoli, L Villa, C Pezzella, E Bordi, P Visca

**Affiliations:** Istituto Superiore di Sanità, Rome, Italy. alecara@iss.it

## Abstract

We conducted a 30-year retrospective analysis of IncFI plasmids from Salmonella enterica serotype Typhimurium. These plasmids have been associated with the emergence of epidemic clones of multidrug-resistant Salmonella. Molecular and genetic evidence indicates that IncFI plasmids are evolving through sequential acquisition of integrons carrying different arrays of antibiotic- resistance genes.


### Expanding Drug Resistance through Integron Acquisition by IncFI Plasmids of *Salmonella enterica* Typhimurium

**Alessandra Carattoli,* Laura Villa,* Cristina Pezzella,* Eugenio Bordi,† and Paolo Visca†‡**

*Istituto Superiore di Sanità, Rome, Italy; †National Institute for Infectious Diseases Lazzaro Spallanzani, Rome, Italy; and ‡Università di Roma Tre, Rome, Italy

We conducted a 30-year retrospective analysis of IncFI plasmids from *Salmonella enterica* serotype Typhimurium. These plasmids have been associated with the emergence of epidemic clones of multidrug-resistant *Salmonella*. Molecular and genetic evidence indicates that IncFI plasmids are evolving through sequential acquisition of integrons carrying different arrays of antibiotic-resistance genes.

Multidrug resistance (MDR) in *Salmonella enterica* serotype Typhimurium has substantial public health and economic impact. Recent epidemiologic data highlighted broadening of antibiotic resistance spectra in this serotype (1), along with worldwide distribution of MDR *S.* Typhimurium clones (2). These clones may have been selected by indiscriminate use of antimicrobial agents in clinical practice and animal husbandry. Understanding the genetic mechanism(s) responsible for acquisition and spread of antibiotic resistance could facilitate development of effective prevention and control strategies.

Acquisition of mobile genetic elements encoding multiple antibiotic resistance genes is the main mechanism for short-term accumulation of resistance determinants in bacterial genomes. Resistance (R-) plasmids of the IncFI incompatibility group have been associated with emergence of MDR *S. enterica* serotypes (3). IncFI plasmids were found most often in Middle Eastern isolates of *S.* Typhimurium, but similar plasmids were also identified in *Salmonella* serotypes from Africa, Europe, and North America (3). Convincing epidemiologic and genetic data support the view that acquisition of IncFI plasmids contributed in the mid-1970s to epidemic spread of *S. enterica* serotype Wien. The IncFI-carrying salmonellae were responsible for protracted outbreaks of severe pediatric infections that were difficult to treat because of the wide spectrum of antimicrobial resistance (4). Most of IncFI plasmids isolated in the early 1970s from epidemic *S.* Typhimurium and *S.* Wien strains conferred resistance to ampicillin (Ap), chloramphenicol (Cm), streptomycin (Sm), spectinomycin (Sp), sulphonamides (Su), tetracycline (Tc), mercuric ions (Hg), and occasionally kanamicin (Km) (3). A few IncFI plasmids, particularly those from Mediterranean *S.* Wien isolates, lacked R-SmSpSu determinants (5). After the *S.* Wien epidemics waned in the early 1980s, outbreaks linked to IncFI-carrying *Salmonella* strains have occasionally been reported (6).

Address for correspondence: Alessandra Carattoli, Laboratory of Bacteriology and Medical Mycology, Istituto Superiore di Sanità, Rome, Viale Regina Elena 299, 00161 Rome, Italy; phone: 39-06-4990-3128; fax: 39-06-4938-7112; e-mail: alecara@iss.it

We have recently shown that 37 MDR *S.* Typhimurium strains isolated from 1996 to 1997 from sporadic cases of pediatric gastroenteritis harbored conjugative IncFI plasmids of around 120 kilobases (kb) with the common profile R-ApCmKmSmSuTp (7). Conjugal transfer of a prototypic IncFI plasmid (designated IncFI/97) from the type strain ST366 to *Escherichia coli* K-12 was associated with transmission of the entire resistance cluster. We demonstrated that most of the antibiotic-resistance genes in this plasmid were located in integrons (7). These genetic elements encode a site-specific recombinase (integrase) capable of capturing resistance genes, which are assembled as gene cassettes controlled by the strong promoter *P*ant. Gene cassettes are flanked by two conserved segments, the 5'CS and 3'CS, encoding the integrase (*intI1*) and the sulphonamide resistance determinant (*sul1*), respectively (8).

Two integrons were identified in IncFI/97: In-t1 (R-CmKmSu), carrying the *aadB* and *catB3* genes, and In-t2 (R-ApSmSu), carrying the *oxa1* and *aadA1* genes (7). Integrons are now recognized as the main genetic vehicles of antibiotic resistance in gram-negative bacteria (9), but it remains unclear to what extent these elements contributed to the expansion of the antibiotic resistance repertoire of IncFI plasmids.

### The Study

To gain insight into the evolution of the resistance determinants on IncFI plasmids, we compared the R-profile and the integron content of IncFI/97 with that of five IncFI plasmids representative of those identified in epidemic *S.* Typhimurium and *S.* Wien from human sources during 1970-1978 (Table). Prototypic plasmids were transferred to *E. coli* K-12 either by conjugation (11) or conventional electrotransformation before antibiotic-susceptibility testing by the disc-diffusion method (12). All but one plasmid showed a common core of antibiotic resistance (R-ApCmTcHgKm), while additional resistance to Sm, Sp, and Su was observed in plasmids IncFI/97, NTP101, pZM3, and TP181.

The IncFI collection was screened for the presence of the integrase gene *intI1*. Six IncFI plasmids were purified from

Table. Designation and relevant characteristics of representative IncFI plasmids used for genetic analysis of integrons

| No. | Plasmid | Host strain | Origin, year[a] | Resistance pattern[b] | Tra[c] | kb | Reference |
|---|---|---|---|---|---|---|---|
| 1 | IncFI/97 | ST 366 | Albania, 1997 | Ap Cm Sm Sp Su Tc Hg Km Tp | + | 120 | 7 |
| 2 | NTP101 | ST DT 208 | England, 1974 | Ap Cm Sm Sp Su Tc Hg | - | 135 | 3 |
| 3 | pZM3 | SW 20 | Algeria, 1970 | Ap Cm Sm Sp Su Tc Hg Km | + | 165 | 10 |
| 4 | TP181 | ST DT 208 | Iran, 1975 | Ap Cm Sm Sp Su Tc Hg Km | + | 165 | 3 |
| 5 | pZM111 | SW WZM111 | Italy, 1978 | Ap Cm Hg Km Gm | + | 130 | 5 |
| 6 | PZM61 | SW WZM6 | Italy, 1974 | Ap Cm Tc Hg Km | - | 145 | 11 |

[a] Year of isolation.
[b] Abbreviations: Ap = ampicillin; Cm = chloramphenicol; Sm = streptomycin; Sp = spectinomycin; Su = sulfonamides; Tc = tetracycline; Hg = mercuric ions; Km = kanamycin; Tp = trimethoprim; Gm = gentamicin.
[c] Autotransferring properties.

*E. coli* K-12 recipients (13), analyzed by agarose gel electrophoresis (Figure 1A), and hybridized with a Tn*21*-derived *intI1* probe. The class 1 integrase gene(s) was located on IncFI plasmids carrying the R-SmSpSu determinants, including isolates from the 1970s as well as more recent ones (Figure 1B). This is the first demonstration of integron presence in MDR *Salmonella* isolates traced to the early 1970s.

To define the structure of integrons carried by IncFI plasmids, Southern blot hybridization of *PvuII-BamHI*-digested DNA with the *intI1* probe was performed. The In-t2 integron was conserved in all the integrase-positive plasmids, including IncFI/97, NTP101, pZM3, and TP181 (Figure 1C). This observation indicates that In-t2 has been maintained unaltered in IncFI plasmids for nearly 30 years. IncFI/97 is likely to be a new plasmid variant, since it contains a second integron, designated In-t1 (7), which appears as a smaller band on the hybridization profile (Figure 1C). Sequence data confirmed the structural organization of the integrons (Figure 1D).

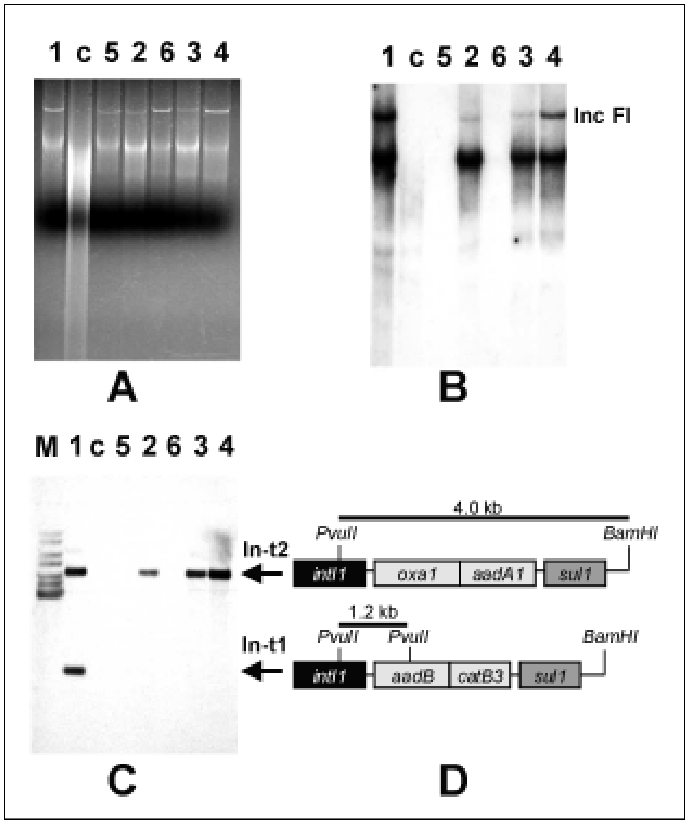

Figure 1. Structural organization of integrons carried by IncFI plasmids. Numbers above each lane indicate plasmid reference numbers as defined (Table). Lane C shows the plasmidless *E. coli* K-12 strain CSH26 (11). A: agarose (0.8%) gel electrophoresis in 1x Tris-borate-EDTA buffer of plasmid DNA extracted from *E. coli* K-12 exconjugants. DNA was stained with ethidium bromide and visualized under UV light. B: Southern blot hybridization of plasmids shown in panel A with the *intI1* probe. C: Southern blot hybridization with the *intI1* probe of *PvuII-BamHI* double-digested DNA from *E. coli* K-12 exconjugants. The DNA was extracted as described (7). The *intI1* probe was amplified with primers corresponding to nt 4680-4700 and nt 5252-5232 of the released Tn*21* sequence (GenEMBL accession no. AF071413). Plasmid pACYC184::Tn*21* (10) was used as the template. The probe was labeled with [α-$^{32}$P]dCTP by using a random priming kit (GibcoBRL). Chromosomal DNA fluoresced because of weak trapping of partially degraded plasmids. D: schematic representation of In-t2 and In-t1 integrons. The thick bars above each structure represent the DNA fragments recognized by the *intI1* probe. The 4.0-kb *PvuII-BamHI* fragment (In-t2) encompasses the *intI1* gene segment downstream of the conserved *PvuII* site (0.6 kb), the *oxa1* and *aadA1* gene cassettes (2.2 kb), and the 3'-CS comprising the *sul1* gene upstream of the *BamHI* site (1.2 kb) (7,8). The 1.2-kb *PvuII-BamHI* fragment (In-t1) encompasses the *intI1* gene segment and part of the *aadB* gene cassette (7).

To further explore the structural identity between recent and ancestral IncFI plasmids, restriction enzyme analysis of plasmid DNAs was performed with *Sal*I and *Sph*I endonucleases. Based on electrophoretic patterns (Figure 2A), moderate similarity between IncFI/97 and IncFI plasmids isolated in the early 1970s can be recognized. The presence of similar-sized restriction fragments is particularly evident for plasmids carrying the In-t2 integron, including IncFI/97, NTP101, pZM3, and TP181. This finding suggests that the evolution of MDR-IncFI plasmids may have occurred through the sequential acquisition of integrons.

To investigate the potential inheritance of genetic elements from ancestral to recent IncFI plasmids, we probed our collection for the presence of additional resistance determinants. Plasmid pZM3 was previously described to contain an IS*15*-like composite element (IS*1936*-Km$^r$) conferring Km-resistance (10). This structure is located within a Tn*21* derivative (Tn*1935*), which also carries an Ap-resistance (*oxa-1*) gene upstream of the resident Sm-resistance (*aadA1*) gene of Tn*21*, in addition to the distal Hg-resistance determinant (10). Although the manner by which new antibiotic resistance genes are acquired by Tn*21*-like transposons was unclear at the time pZM3 was characterized (10), acquisition of *oxa-1* likely occurred by integrase-mediated recombination. This event led to the assembly of In-t2 from its putative precursor, the Tn*21*-borne In2 integron

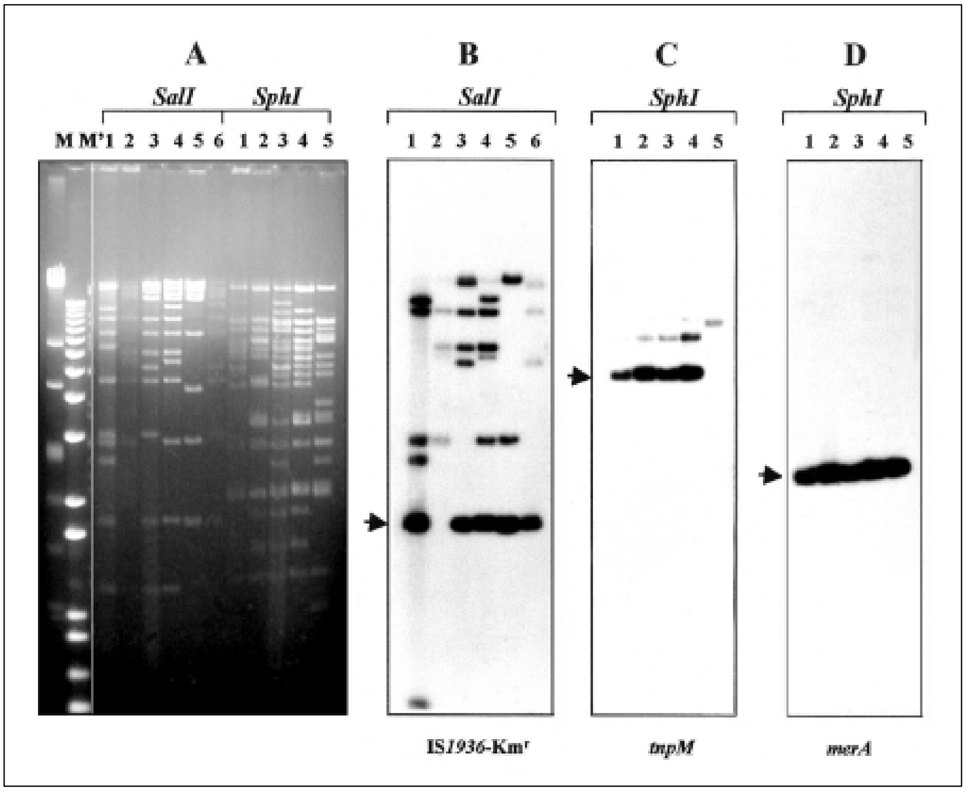

Figure 2. Restriction enzyme analysis and detection of common genetic elements in IncFI plasmids. Numbers above each lane indicate plasmid reference numbers as defined (Table). M and M' are *Hin*dIII-digested lambda DNA and 1-kb plus DNA ladder (GibcoBRL), respectively. A: agarose (0.8%) gel electrophoresis in 1x Tris-borate-EDTA buffer of plasmids digested with restriction endonucleases *SalI* and *SphI*. DNA was stained with ethidium bromide and visualized under UV light. B: Southern blot hybridization of *SalI*-digested plasmids with the IS*1936*-Km$^{r}$ probe purified as 1.8-kb *SalI* fragment from plasmid pACYC184::Tn*1935* (10). C and D: Southern blot hybridizations of *SphI*-digested plasmids with the *tnpM* and *merA* probes, respectively. The *tnpM* probe was amplified with primers corresponding to nt 3689-3709 and nt 4070-4050 of the released Tn*21* sequence (GenEMBL accession no. AF071413). For synthesis of the *merA* probe, primers annealing to nt 17224-17249 and nt 17931-17907 of the Tn*21* sequence were used. Plasmid pACYC184::Tn*21* (10) was used as the template. Probes were labeled with [$\alpha$-$^{32}$P]dCTP by using a random priming kit (GibcoBRL, Rockville, MD). The arrows point to predicted hybridization fragments.

(9). Therefore, we tested IncFI plasmids for the presence of the IS*1936*-Km$^{r}$ element, the transposase (*tnp*) machinery, and the Hg-resistance (*mer*) locus. All but one of the IncFI plasmids carry the IS*1936*-Km$^{r}$ element (Figure 2B). NTP101 is the only exception, consistent with the Km-susceptible phenotype conferred by this plasmid. Additional bands, some of which were shared by different IncFI plasmids, were also detected, likely as a result of cross-hybridization of the probe with IS*15*-like elements located on these replicons. Plasmids IncFI/97, NTP101, PZM3, and TP181 were also positive for the presence of the 4.4-kb *SphI* band (Figure 2C), which is predicted to contain the *tnpM* (regulator), the *tnpR* (resolvase), and part of the *tnpA* (transposase) genes of Tn*21*-like transposons (GenEMBL accession no. AF071413).

Furthermore, the IncFI plasmids recognized by the *merA* probe also showed a common *SphI* band of 2.1 kb, providing evidence for a structural conservation of the *mer* operon (Figure 2D). Plasmid pZM61 showed hybridization patterns indistinguishable from those of pZM111 (data not shown).

Taken together, the above data indicate that IncFI/97 shares antibiotic resistance determinants with the ancestor plasmids pZM3, NTP101, and TP181, suggesting that MDR developed through independent acquisition of integrons within the structurally conserved IncFI plasmid scaffold. The evolutionary story of these plasmids combines the maintenance of indigenous antibiotic resistance genes with the acquisition of new determinants, leading to accumulation of multiple resistance mechanisms. For example, the *oxa1* gene cassette was acquired despite the presence of the TEM-type beta-lactamase encoded by the preexisting Tn*A* transposon (11). Likewise, acquisition of In-t1 introduced a third aminoglycoside-resistance determinant (*aadB*) in addition to the resident IS*1936*-Km$^{r}$ (*aph*) element (10) and the In-t2-encoded *aadA1* gene. Along the same lines, resistance to chloramphenicol was determined by both the *catB3* gene cassette of In-t1 and by the resident *cmlA* gene. Besides increasing the level of antibiotic resistance due to the expression of multiple detoxification mechanisms, acquisition of new resistance genes is also expected to enlarge the spectrum of resistance. Our results (not shown) indicate that In-t2 serves as a vehicle for the *oxa-1* gene, extending beta-lactam resistance to ureidopenicillins, and of the *aadA1* gene, conferring resistance to streptomycin and spectinomycin. Likewise, the In-t1-carried *aadB* gene broadens the aminoglycoside resistance to tobramycin and gentamicin.

### Conclusions

Our retrospective investigation provides novel evidence for the involvement of integrons in the development of MDR through sequential acquisition of new resistance determinants within IncFI plasmids. This family of replicons could play a role in the future development of resistance in *S.* Typhimurium and, more generally, in Enterobacteriaceae. A dramatic outcome of this evolutionary pathway may be predicted from the intrinsic structural and functional properties of mobile DNA elements found on these R-plasmids. Integrons provide a unique mechanism for the recruitment of additional resistance genes. Moreover, spreading resistance genes among different replicons is favored by the location of integrons within transposable elements. Finally, horizontal transmission is expected because of the conjugative or mobilizable properties of IncFI carriers, which enables them to traverse species and genus boundaries. Future success in controlling resistance depends, at least in part, on a complete understanding of the mechanisms involved in dissemination. A systematic search for integrons and transposable elements could provide a useful genetic basis for MDR in human and animal *S.* Typhimurium isolates.

### Acknowledgments

We thank B. Colonna, M.A. Casalino, and M. Nicoletti for the encouraging discussion and the generous gift of strains and plasmids and F. Angulo, S. Fanning, and R. Helmuth for critical reading of the manuscript and helpful suggestions.

This investigation was supported by grants from the Italian Ministry of Health to AC and PV.

Dr. Carattoli is a senior scientist at the Reference Center for Pathogenic Enterobacteria of Istituto Superiore di Sanità. Her research interest focuses on the genetic basis of multidrug resistance and pathogenesis in gram-negative bacteria.

### References

1. Glynn MK, Bopp C, Dewitt W, Dabney P, Mokhtar M, Angulo FJ. Emergence of multidrug-resistant *Salmonella enterica* serotype Typhimurium DT104 infections in the United States. N Engl J Med 1998;338:1333-8.
2. Prager R, Liesegang A, Rabsch W, Gericke B, Thiel W, Voigt W, et al. Clonal relationship of *Salmonella enterica* serovar Typhimurium phage type DT104 in Germany and Austria. Zentralb Bakteriol 1999;289:399-414.
3. Anderson ES, Threlfall EJ, Carr JM, McConnell MM, Smith HR. Clonal distribution of resistance plasmid-carrying *Salmonella typhimurium*, mainly in the Middle East. J Hyg (Lond) 1977;79:425-48.
4. Domart A, Robineau M, Stroh A, Dubertret LM, Modai J. Septicemia due to *Salmonella wien*. Diagnostic, therapeutic and epidemiologic problems. Ann Med Interne (Paris) 1974;125:915-8.
5. Casalino MA, Comanducci A, Nicoletti M, Maimone F. Stability of plasmid content in *Salmonella wien* in late phases of the epidemic history. Antimicrob Agents Chemother 1984;25:499-501.
6. Frost JA, Rowe B, Ward LR, Threlfall EJ. Characterization of resistance plasmids and carried phages in an epidemic clone of multi-resistant *Salmonella typhimurium* in India. J Hyg (Lond) 1982;88:193-204.
7. Tosini F, Visca P, Luzzi I, Dionisi AM, Pezzella C, Petrucca A, Carattoli A. Class 1 integron-borne multiple-antibiotic resistance carried by IncFI and IncL/M plasmids in *Salmonella enterica* serotype Typhimurium. Antimicrob Agents Chemother 1998;42:3053-8.
8. Hall RM, Collis CM. Mobile gene cassettes and integrons: capture and spread of genes by site-specific recombination. Mol Microbiol 1995;15:593-600.
9. Liebert CA, Hall RM, Summers AO. Transposon Tn*21*, a flagship of the floating genome. Microbiol Mol Biol Rev 1999;63:507-22.
10. Colonna B, Bernardini M, Micheli G, Maimone F, Nicoletti M, Casalino M. The *Salmonella wien* virulence plasmid pZM3 carries Tn*1935*, a multiresistance transposon containing a composite IS*1936*-kanamycin resistance element. Plasmid 1988;20:221-31.
11. Maimone F, Colonna B, Bazzicalupo P, Oliva B, Nicoletti M, Casalino MA. Plasmids and transposable elements in *Salmonella wien*. J Bacteriol 1979;139:369-75.
12. National Committee for Clinical Laboratory Standards. Performance standards for antimicrobial disc susceptibility tests. M2A2. Villanova (PA): The Committee; 1992.
13. Kado CI, Liu S. Rapid procedure for detection and isolation of large and small plasmids. J Bacteriol 1981;145:1365-73.
